# RGB Channel Superposition Algorithm with Acetowhite Mask Images in a Cervical Cancer Classification Deep Learning Model

**DOI:** 10.3390/s22093564

**Published:** 2022-05-07

**Authors:** Yoon Ji Kim, Woong Ju, Kye Hyun Nam, Soo Nyung Kim, Young Jae Kim, Kwang Gi Kim

**Affiliations:** 1 Department of Biomedical Engineering, Gil Medical Center, College of Medicine, Gachon University, 21 Namdong-daero 774 Beon-gil, Namdong-gu, Incheon 21565, Korea; younji524@gachon.ac.kr (Y.J.K.); youngjae@gachon.ac.kr (Y.J.K.); 2 Department of Obstetrics & Gynecology, Seoul Hospital, Ewha Womans University, Seoul 07804, Korea; goodmorning@ewha.ac.kr; 3 Department of Obstetrics & Gynecology, Bucheon Hospital, Soonchunhyang University, Bucheon-si 14584, Korea; khnam@schmc.ac.kr; 4 R & D Center, NTL Medical Institute, Seongnam-si 13449, Korea; snkim@chollian.net; 5 Department of Health Sciences and Technology, Gachon Advanced Institute for Health Sciences and Technology (GAIHST), Gachon University, Incheon 21565, Korea

**Keywords:** cervical cancer, acetowhite, RGB channel superposition, deep learning, ResNet

## Abstract

Cervical cancer is one of the main causes of death from cancer in women. However, it can be treated successfully at an early stage. This study aims to propose an image processing algorithm based on acetowhite, which is an important criterion for diagnosing cervical cancer, to increase the accuracy of the deep learning classification model. Then, we mainly compared the performance of the model, the original image without image processing, a mask image made with acetowhite as the region of interest, and an image using the proposed algorithm. In conclusion, the deep learning classification model based on images with the proposed algorithm achieved an accuracy of 81.31%, which is approximately 9% higher than the model with original images and approximately 4% higher than the model with acetowhite mask images. Our study suggests that the proposed algorithm based on acetowhite could have a better performance than other image processing algorithms for classifying stages of cervical images.

## 1. Introduction

Cervical cancer is the fourth most common cancer in women worldwide. In 2018, approximately 570,000 women were diagnosed with cervical cancer, and an estimated 311,000 women, representing 7.5% of all female cancer deaths, died due to cervical cancer worldwide [1]. Cervical cancer is divided into two large groups, namely, atypical (A) and positive (P), which is the precancerous stage. A is graded into atypical 1 (A1) and atypical 2 (A2), and P, for human papillomavirus-infected tissue in which cervical cancer progresses, is classified as positive 1A (P1A), positive 1 B (P1B), and positive 2 (P2) depending on the state of lesions [2]. Cervical cancer is one of the cancers that can be treated successfully, as long as it is detected early and managed effectively [3,4,5]. Therefore, early diagnosis through regular screening is important. It is important to distinguish between A and P because treatment is required when the patient is in case P, which is the initial state of the lesion. Furthermore, when analyzing actual cases, A1 shows an overwhelming number of atypical cases, and P1B shows the largest number of dysplasia cases [6]. Therefore, a clear distinction between A1 and P1B cases is necessary.

Tests for the early diagnosis of cervical cancer include cervical Pap smear, colposcopy, and cervicography. A Pap smear test, which is one of the existing methods for diagnosing cervical cancer, is based on cytology and requires electricity for a microscope, consumables for examination, and experts for the interpretation of results, making it difficult to be implemented in an environment with insufficient resources [7]. In addition, the sensitivity estimate for the detection of invasive cancer is low, so repeated screening tests are required [8]. To compensate for the high false-negative rate of the Pap smear test, colposcopy was performed to diagnose lesions by directly observing the changed cervix after applying 3–5% acetic acid. Colposcopy shows a high accuracy, but depending on the experience of the specialist performing the examination, small lesions may not be detected and may be omitted, resulting in different results [9,10]. Cervicography is a test that diagnoses the lesion by enlarging the image after taking a picture of the cervix coated with acetic acid. This test has the advantage of being simple and maintaining objectivity [11]. However, technical defects may occur due to obstruction of the visual field, and a relatively high false-positive rate due to metaplasia, etc. [12,13].

When it is difficult to distinguish between normal and lesions in all cervical cancer diagnostic tests, one of the important abnormal findings to pay attention to in lesion diagnosis is the appearance of white spots on the cervix after acetic acid application in cervicography, that is, the expression of acetowhite [14,15,16,17]. The density of acetowhite areas generally increases with lesion severity. Clear and thin acetowhite areas are most likely due to immature metaplasia or inflammation, and thin but opaque areas of acetowhite are more likely to be asymptomatic for papillomavirus infection (SPI) or CIN1. Distinct opaque acetowhite areas after acetic acid application suggest high-grade lesions (HSILs). In addition, if the edge of the acetowhite region is unclear or angled, then it can be determined as a metaplasia, SPI, or CIN1, and the more regular the boundary of the acetowhite region, the more likely the HSIL is [18].

Because acetowhite is an important criterion for diagnosing lesions according to its color and shape, it is gaining attention as a tool to overcome the limitations of the accuracy and diagnostic efficiency of existing colposcopy [19]. In 2015, Sheng et al. showed an average of 0.7765 DSC by eliminating unbalanced labels through class-averaging graph-based transduction based on superpixels made with the *k*-means clustering algorithm [20]. In addition, in 2021, Yue et al. performed acetowhite segmentation with a 0.835 dice coefficient and 93.40% precision using a deep attention network for an image to which the specular reflection removal algorithm was applied [21]. In 2021, Liu et al. extracted the cervical region using the *k*-means clustering algorithm and then segmented acetowhite with an accuracy of 90.36% using a pre-trained ResNet101-based DeepLab V3+ network [22]. To date, deep learning has been used as a tool for the automatic segmentation of acetowhite [23,24,25]. However, to the best of our knowledge, no classification model based on acetowhite has been reported.

Therefore, in this paper, we compared the performance of the model for the original image without image processing, the mask image with acetowhite as the region of interest, and the RGB channel superposition image using the original image and mask image. In conclusion, we propose an image processing algorithm that accurately classifies images of the A1 and P1B states, which is a boundary between normal and abnormal, through RGB channel superposition using the acetowhite mask image. This method made it possible to intensively learn about the characteristics of acetowhite according to the state of each lesion.

## 2. Methods

### 2.1. Data

The data in this study consisted of simple atypical A1 and dysmorphic P1B images, and 438 A1 and 477 P1B images were used, respectively. Of the cervical images, 670 were obtained using the Dr. Cervicam+ camera (https://ntlhealthcare.com (accessed on 17 March 2021), Seongnamsi, Korea), and 245 were obtained using the Dr. Cervicam camera (https://ntlhealthcare.com (accessed on 17 March 2021), Seongnamsi, Korea). As for the age composition of patients, 190 of 915 patients were sampled and surveyed, and 46.32% were in their 20s or younger, 28.95% in their 30s, 17.37% in their 40s, and 7.37% in their 50s or older. The training set used for training the deep learning model and the test set used for evaluating the deep learning model consisted of a ratio of 8:2. The training set comprised 350 A1 and 382 P1B, and the test set comprised 108 A1 and 95 P1B.

### 2.2. Image Preprocessing

For the collected cervical images, an expert directly annotated the acetowhite as a rectangular type using the NTL AI Data Manager system (https://ntlhealthcare.com (accessed on 23 May 2021), Seongnamsi, Korea). Except for the annotated area, the background was treated with black to create a mask image of the acetowhite area. The captured cervical images were all constant at 1504 × 1000 pixels. Except for the external os located in the center of the image, the vaginal wall and colposcopy were on both sides of the image, so unnecessary parts for learning were included. Accordingly, both sides were cropped based on the center such that the aspect ratio of all images was the same, and the size was converted to 256 × 256 pixels. To generate an RGB channel superposition image, the acetowhite mask image and original image were prepared by separating each RGB channel image.

### 2.3. RGB Channel Superposition

The RGB channel superposition image clearly shows the acetowhite region of interest, while also learning the pixel values of the acetowhite periphery.

**Figure 1:**
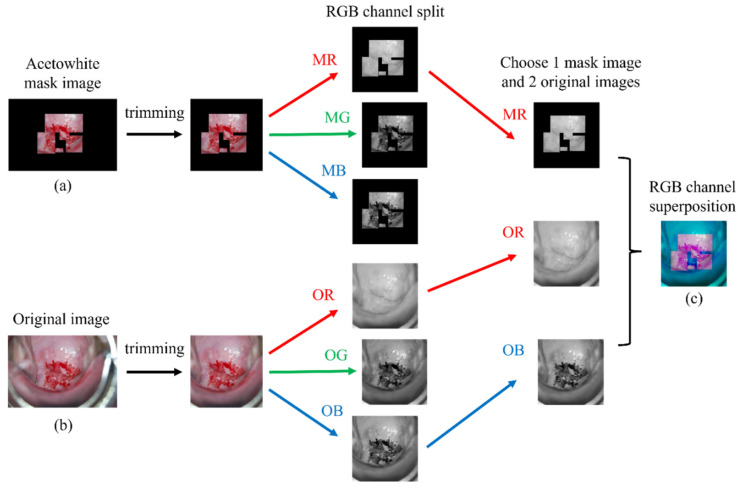
A schematic diagram of the RGB channel superposition process. (**a**) Acetowhite mask image (**b**) Original image (**c**) RGB channel superposition image.

To create an RGB channel superposition image, the original image was divided into R, G, and B channels to have one channel and defined as OR, OG, and OB, respectively. In addition, the acetowhite mask image created through image preprocessing was divided into images with one channel and defined as MR, MG, and MB, respectively. The RGB channel superposition image was created by selecting two channels from the three channels of the original image, selecting one channel from the three channels of the mask image, and placing each image into the three RGB channels and merging them. Figure 1 shows a schematic of the RGB channel superposition process.

**Figure 2:**
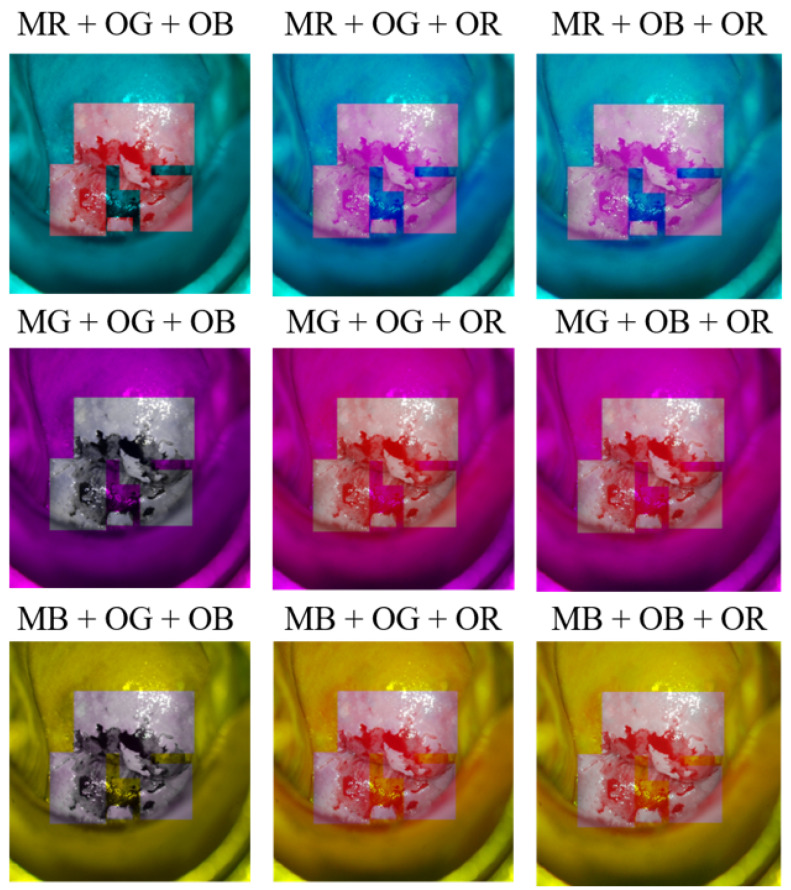
Nine cases of cervical images made through RGB channel superposition.

For example, if MR is selected as one of the three channels of the mask image and OG and OB are selected as two of the three channels of the original image, the acetowhite part has a purple-red color, and in the other areas, a blue-colored image is created. Because one image has three RGB channels, if multiple cases are created by selecting one of the three channels of the mask image and two of the three channels of the original image in the same way, a total of nine cases will be made. Figure 2 shows each of the nine cases created by the RGB channel superposition.

Each RGB channel superposition image consisted of 438 A1 and 477 P1B images identical to the original image and was used for the training and testing of each model. The OpenCV library (version 4.5.0) was used to superpose the RGB channels of the original image and acetowhite mask image.

### 2.4. Classification Deep Learning Model

**Figure 3:**
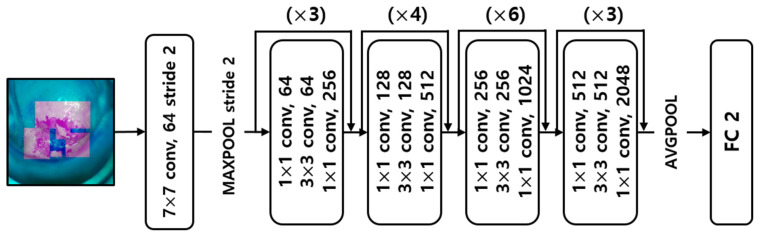
Diagram of the ResNet 50 model architecture.

The image classification deep learning model used in this study is the ResNet. Based on the VGGNet, a shortcut is placed between the convolutional layers, and the input value *x* was added to the output value *F*(*x*) after the training layer to determine the minimum value of *F*(*x*) + *x* and use it for the next input value. Thus, by learning the optimal *F*(*x*) + *x*, the classification performance increases as the layers become deeper, and the error rate is lower than that of VGGNet or GoogLeNet [26]. ResNet 50 is a model with 50 convolutional layers in the ResNet structure. Figure 3 shows the ResNet 50 model structure with shortcuts connected every three layers.

A learning process that requires a large amount of data and time is essential for a deep-learning model to achieve a high level of performance. Therefore, transfer learning was used, which enables high performance with a small amount of data by learning and training the prepared data based on the weights of the pretrained model [27,28]. In this study, a ResNet 50 model based on ImageNet was trained using the Adam optimizer; the batch size was 40, the epoch was 200, and the learning rate was set to 0.0001.

### 2.5. Evaluation of the Deep Learning Model Performance

In this study, to evaluate the performance of the deep learning classification model, the precision and recall, F1-score and accuracy, and area under curve (AUC) score were calculated by comparing the ground truth of the data and the deep learning classification results.

True negative (TN) designates cases when a normal cervical image was classified as normal, whereas true positive (TP) represents cases when a lesioned cervical image was classified as an abnormal. A case in which a normal cervical image was classified as abnormal is defined as a false positive (FP), and a case in which the abnormal cervical image was classified as normal is defined as a false negative (FN). The four indicators used to evaluate the performance of the deep learning classification model were calculated using Equations (1)–(4).

(1) $$\mathrm{Precision}\;=\frac{\mathrm{TP}}{\mathrm{TP}+\mathrm{FP}}\times 100$$

(2) $$\mathrm{Recall}\;=\frac{\mathrm{TP}}{\mathrm{TP}+\mathrm{FN}}\times 100$$

(3) $$F1-\mathrm{score}\;=\frac{\mathrm{Precision}\;\times \;\mathrm{Recall}}{\mathrm{Precision}\;+\;\mathrm{Recall}}\times 2$$

(4) $$\mathrm{Accuracy}\;=\frac{\mathrm{TP}+\mathrm{TN}}{\mathrm{TN}+\mathrm{TP}+\mathrm{FP}+\mathrm{FN}}\times 100$$

In addition, a receiver operating characteristic (ROC) curve for the performance of each model was drawn, and AUC, which is the area under the ROC curve, was calculated. The closer the AUC is to 1, the better the model’s performance.

### 2.6. Statistical Analysis

Statistical analysis was performed to confirm the statistical significance between the study results using MedCalc (version 8.2.1.0, MedCalc Software, Ostend, Belgium). The precision, recall, F1-score, accuracy, and AUC of the original image model, acetowhite mask model, and RGB channel superposition model were compared and analyzed using the Friedman–Nemenyi test. A *p*-value less than 0.05 is considered statistically significant. For the RGB channel superposition model, the model showing the highest performance among the nine models was selected, and the statistical significance of the results was checked. We further confirmed the statistical significance using the critical difference diagram which shows that the mean ranks of each model under 5 different deep learning model performance evaluation methods. The lower the rank, further to the left, the better the performance of a model compared to the others [29].

## 3. Results

The precision, recall, F1-score, accuracy, and AUC were calculated to evaluate the classification performance of each model, to which the original images, acetowhite mask images, and RGB channel superposition images were trained. To prevent overfitting and increase the reliability of the deep learning model performance evaluation, the entire dataset was divided into five and evaluated through five cross-validations using each as a test set once. Table 1 shows the average deep learning model performance evaluation score of each RGB channel superposition case calculated through five cross-validations.

The original image model showed a precision of 84.73%, recall of 57.45%, F1-score of 68.25%, and accuracy of 72.46%. In the model trained on the image made with the mask using acetowhite as the region of interest, the precision was 84.70%, recall rate was 66.45%, the F1-score was 74.41%, and the accuracy was 76.28%. In the model trained with the original image and acetowhite mask image, to which the RGB channel superposition algorithm was applied, the model with the highest performance had a precision of 90.05%, a recall rate of 72.55%, an F1-score of 79.94%, and an accuracy of 81.31%. Table 2 shows the deep learning performance evaluation score of each model depending on the applied algorithm.

**Figure 4:**
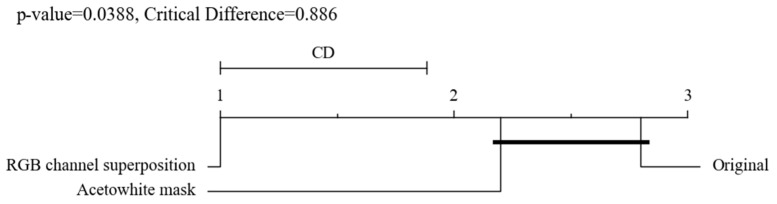
Critical Difference Diagram of the Friedman–Nemenyi test for deep learning model performance comparison. The number shows the lank of three models. The lower the rank, the better the performance of a model.

We compared the precision, recall, F1-score, accuracy, and AUC of the original image model, acetowhite mask model, and RGB channel superposition model using the Friedman–Nemenyi test. The result from the test shows 0.0388 of *p*-value. Figure 4 shows the critical difference diagram of the models.

**Figure 5:**
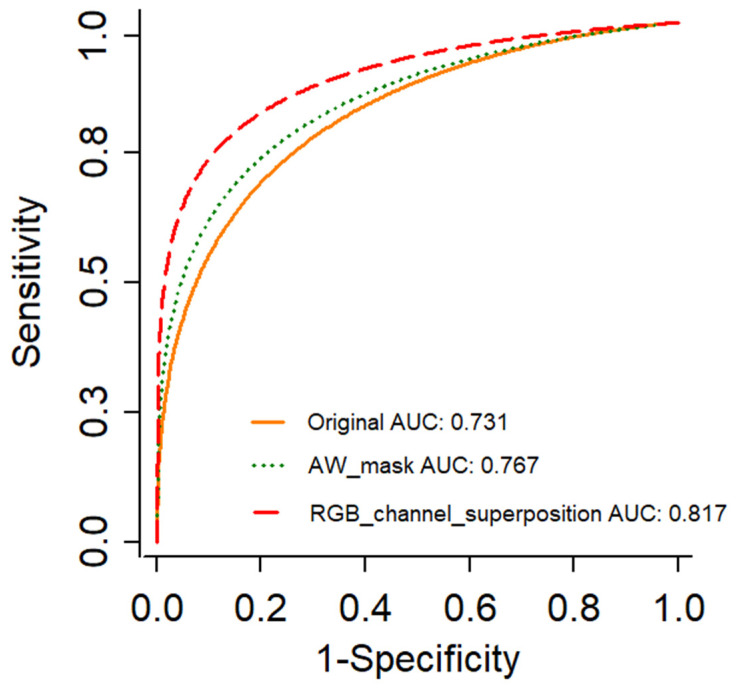
ROC graph and AUC of the original, acetowhite mask, and RGB channel superposition models.

The ROC graph and AUC of the original image model, acetowhite mask model, and RGB channel superposition model are shown in Figure 5. The AUC values were 0.731 in the original image model, 0.767 in the acetowhite mask image model, and 0.817 in the RGB channel superposition model.

## 4. Conclusions

In this study, we compared the performance of a deep learning classification model for cervical cancer, the original image without image processing, a mask image made with acetowhite as the region of interest, and an RGB channel superposition model, which was created by selecting the channel in the original image and acetowhite mask image. We aim to propose an image processing algorithm for improving the classification performance. Based on the evaluation results of the deep learning classification performance, the original image showed 72.46% accuracy and 0.731 AUC, and the acetowhite mask image showed 76.28% accuracy and 0.767 AUC. The acetowhite mask image model showed an improvement of approximately 4% compared with the original image model. The model with the highest performance among the nine cases of RGB channel superposition is the model with the R channel of the acetowhite mask and the R and B channels of the original image. This model showed an accuracy of 81.31% and an AUC of 0.817, which is approximately 9% higher than those of the original image model and approximately 5% higher than those of the acetowhite mask image model.

As a result of the Friedman–Nemenyi test, which can verify the statistical significance, it shows 0.0388 of *p*-value, meaning a statistically significant difference. In addition, the critical difference diagram shows that the leftmost of the three models, the RGB channel superposition model, has the best performance compared to others.

The model trained with the acetowhite mask image had a better performance than the original model because the characteristic of acetowhite, a white spot that appears on the cervix after acetic acid treatment, is an important criterion for diagnosing the stage of the lesion [30]. The mask image made with acetowhite as the region of interest reduces the influence of additional elements, such as the vaginal wall and colposcopy, except for the acetowhite part, and enables a deep learning model to efficiently train the features of acetowhite.

RGB channel superposition is an algorithm that creates an image by taking one channel from the acetowhite mask image and two channels from the original image. The performance of this RGB channel superposition was superior to that of the original or acetowhite mask image. For the acetowhite region of interest, the pixels of all three channels were trained, and for parts other than the region of interest, the pixels of the two channels were trained. It is thought that this is because it uses less data on parts other than the region of interest and uses more information about the acetowhite region of interest for training.

Among the nine models combined with the RGB channel superposition algorithm, the model that showed the highest performance was the one combining the R channel of the acetowhite mask and the R and B channels of the original image. When the histogram of the cervical image was analyzed, the number of pixels in the image was largest in the order of the R, B, and G channels. As a result, this model, which combines the R channel of the mask and the R and B channels of the original image, trained the image with the highest number of pixels among the nine models created by superposing the RGB channels. Accordingly, it had the highest performance among the nine models as it obtained the largest amount of pixel information for acetowhite and peripheral pixel information from the cervical image.

Various methods were proposed to utilize the acetowhite region of interest for deep learning through a systematic comparison of each model. However, a more advanced deep learning classification model can be developed through further research. In this study, a mask was created using the acetowhite region of interest as a rectangular region. However, if a polygonal region mask that can show a clear boundary for acetowhite is created, then the characteristics of the acetowhite boundary can be trained more clearly. It is expected that the impact of areas not necessarily included in the rectangular region can be reduced. In addition, in this study, acetowhite ROI data manually annotated by specialists and experts were used. Therefore, the cervical data are insufficient for training. The classification performance of the deep learning model will be further improved using a sufficient amount of cervical data annotated with acetowhite or data augmentation to satisfy the amount of data required for training.

According to the results of this study, if the RGB channel superposition algorithm is applied to a cervical classification image, the performance of the deep learning model of cervical cancer can be improved by training the acetowhite region with more pixel information than the peripheral part. Therefore, the diagnostic efficiency and accuracy of professional personnel in cervical cancer screening and diagnosis are expected to increase in the future. In addition, it is expected to help develop a CAD system for diagnosing cervical cancer by providing various evaluation indicators for the use of acetowhite in deep learning.

## Figures and Tables

**Table 1:** Deep learning model performance evaluation score of each RGB channel superposition case.

	Precision (%)	Recall (%)	F1-Score (%)	Accuracy (%)
MR + OG+ OB	90.18	68.51	77.51	79.56
MR + OG + OR	89.60	69.59	77.96	79.89
MR + OB + OR	90.05	72.55	79.94	81.31
MG + OG + OB	89.18	70.46	78.61	80.22
MG + OG + OR	89.75	70.03	78.37	80.22
MG + OB + OR	89.86	68.96	77.85	79.67
MB + OG + OB	91.19	68.53	77.58	79.89
MB + OG + OR	87.88	70.65	78.10	79.67
MB + OB + OR	88.77	68.35	77.26	78.80

**Table 2:** Performance evaluation score of each deep learning model according to the applied algorithm.

	Precision (%)	Recall (%)	F1-Score (%)	Accuracy (%)
Original image	84.73	57.45	68.25	72.46
Acetowhite mask	84.70	66.45	74.41	76.28
RGB channel superposition	90.05	72.55	79.94	81.31

## Data Availability

Not applicable.
